# Supplementary material for: Actomyosin forces trigger a conformational change in desmoplakin within desmosomes
Source: Nat Commun. 2025 Oct 10;16:9052. doi: 10.1038/s41467-025-64124-4 (PMC12514153; doi:10.1038/s41467-025-64124-4)
Supplement: Supplementary file 1 — Supplementary Information [file 41467_2025_64124_MOESM1_ESM.pdf]

Supplementary Information

Title: Actomyosin forces trigger a conformational change in desmoplakin within desmosomes

Authors: Yinchen Dong [1], Ahmed Elgerbi [2], Bin Xie [3], Yerin Han [4], Adam V. Kwiatkowski [4], John Choy [2], Sanjeevi Sivasankar [1, 3, *]

Affiliations: [1] Department of Biomedical Engineering, University of California, Davis, CA; [2] Department of Biology, The Catholic University of America, Washington, DC; [3] Biophysics Graduate Group, University of California, Davis, CA; [4] Department of Cell Biology, University of Pittsburgh School of Medicine, Pittsburgh, PA.

***Corresponding author:** ssivasankar@ucdavis.edu

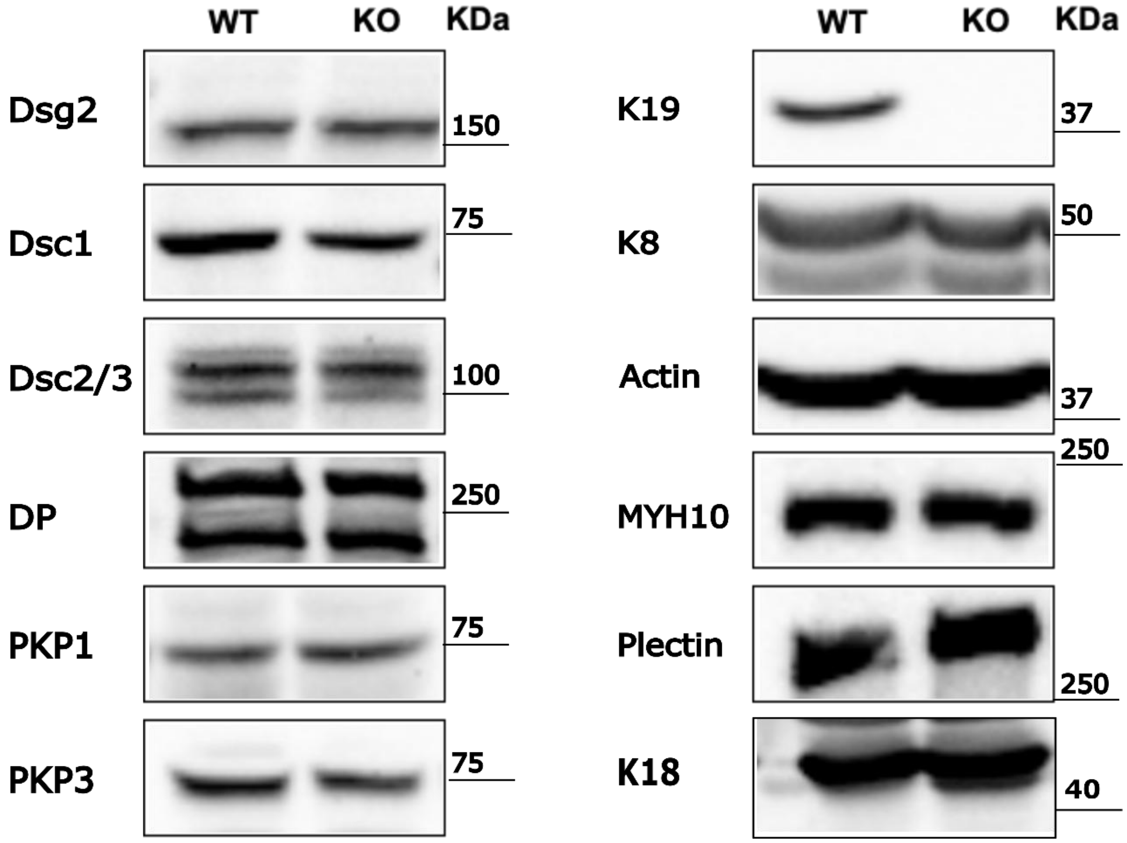

Figure S1. Immunoblot of desmosomal cadherins, plaque proteins, and cytoskeletal proteins. No discernible differences in cadherin, plaque protein, and actomyosin protein levels were detected between WT and K19-KO cells. K19 was present only in WT cells and not in K19-KO cells. Molecular weights from the corresponding protein ladders are marked. Dsg2: Desmoglien-2; Dsc1: Desmocolin-1, Dsc2/3: Desmocolin-2 and Desmocolin-3; DP: Desmoplakin; PKP1: Plakophilin-1; PKP3: Plakophilin-3; K19: Keratin-19; K8: Keratin-8; K18: Keratin-18. Immunoblotting was performed as described in the method section. Each experiment was repeated across three biological replicates. Full uncropped gels are provided in the Source Data file.

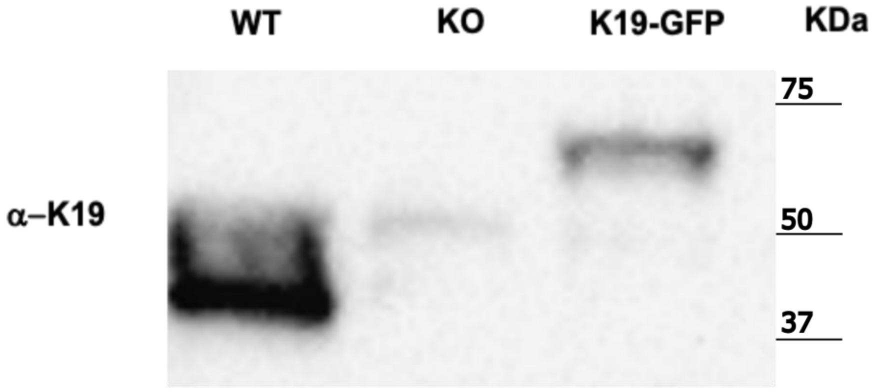

Figure S2. Immunoblot results of K19 in WT, K19-KO, and K19-GFP cells confirm successful re-expression of K19 in the K19-GFP rescue cell line. The K19 band in WT cells is ~ 3x the K19-GFP cells. Molecular weights measured from the ladder are indicated. K19 in WT cells has a molecular weight of ~ 40 kDa, while K19 tagged with GFP in K19-GFP cells has a molecular weight of ~ 70 kDa. Immunoblotting was performed as previously described in the methods section. Each experiment was repeated across three biological replicates. Full uncropped gels are provided in the Source Data file.

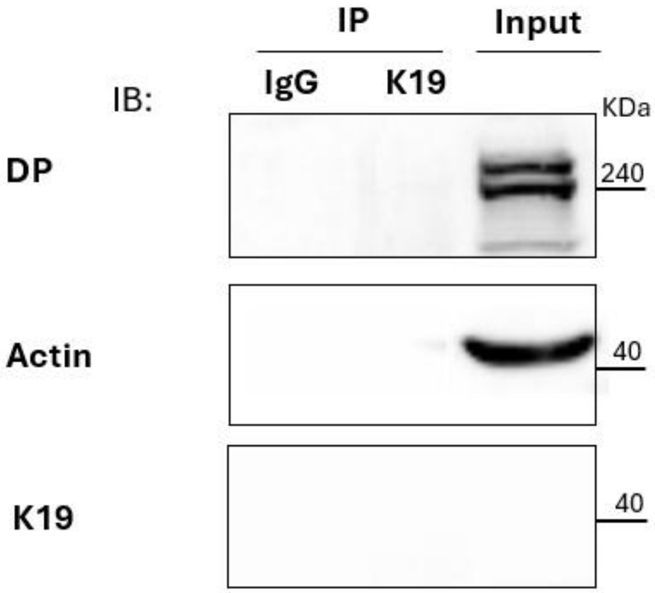

Figure S3. Co-IP of K19 in K19-KO cells. Co-IPs were performed with anti-K19 antibody or IgG control. The co-IPs show that proteins pulled down with K19 in the WT cells were due to specific interactions. Each experiment was repeated across three biological replicates. Full uncropped gels are provided in the Source Data file.

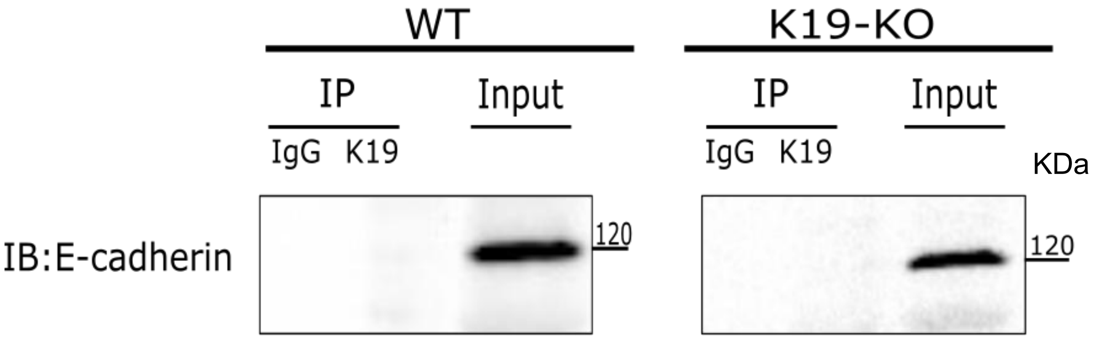

Figure S4. E-cadherin does not co-IP with K19. No E-cadherin bands were pulled down with K19 in both WT and K19-KO cells, serving as an additional negative control to show that only specific interactions were observed for the co-IP results for K19. Each experiment was repeated across three biological replicates. Full uncropped gels are provided in the Source Data file.

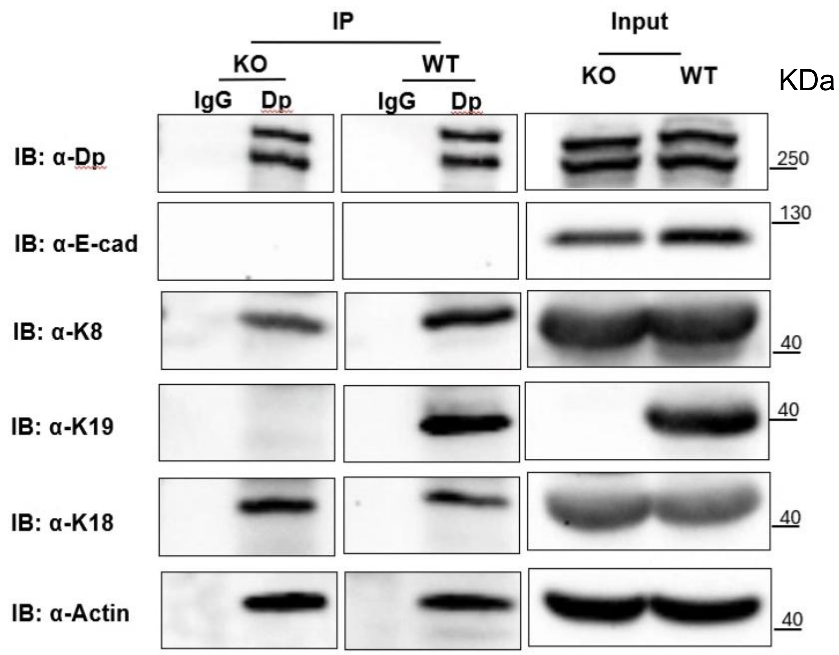

Figure S5. DP co-IPs with keratin and F-actin filaments. Co-IPs were performed using an anti-DP antibody or IgG control in MCF7 WT and K19-KO cells. In WT cells, DP interacts with K8, K18, K19, and F-actin. In K19-KO cells, interactions with K8, K18, and F-actin persist in the absence of K19. Each experiment was repeated across three biological replicates. Full uncropped gels are provided in the Source Data file.

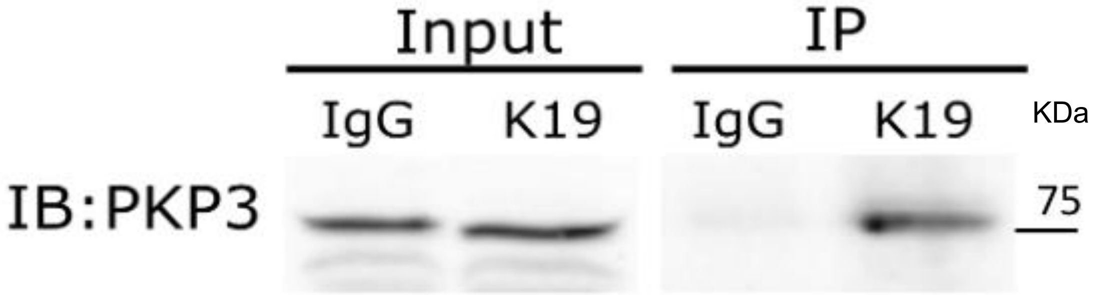

Figure S6. Co-IP shows an interaction between K19 and desmosomal plaque protein PKP3. PKP3 bands were observed for both the IgG control and K19 in the input samples, but the PKP3 band was only observed for the K19 samples in the immunoprecipitation. Each experiment was repeated across three biological replicates. Full uncropped gels are provided in the Source Data file.

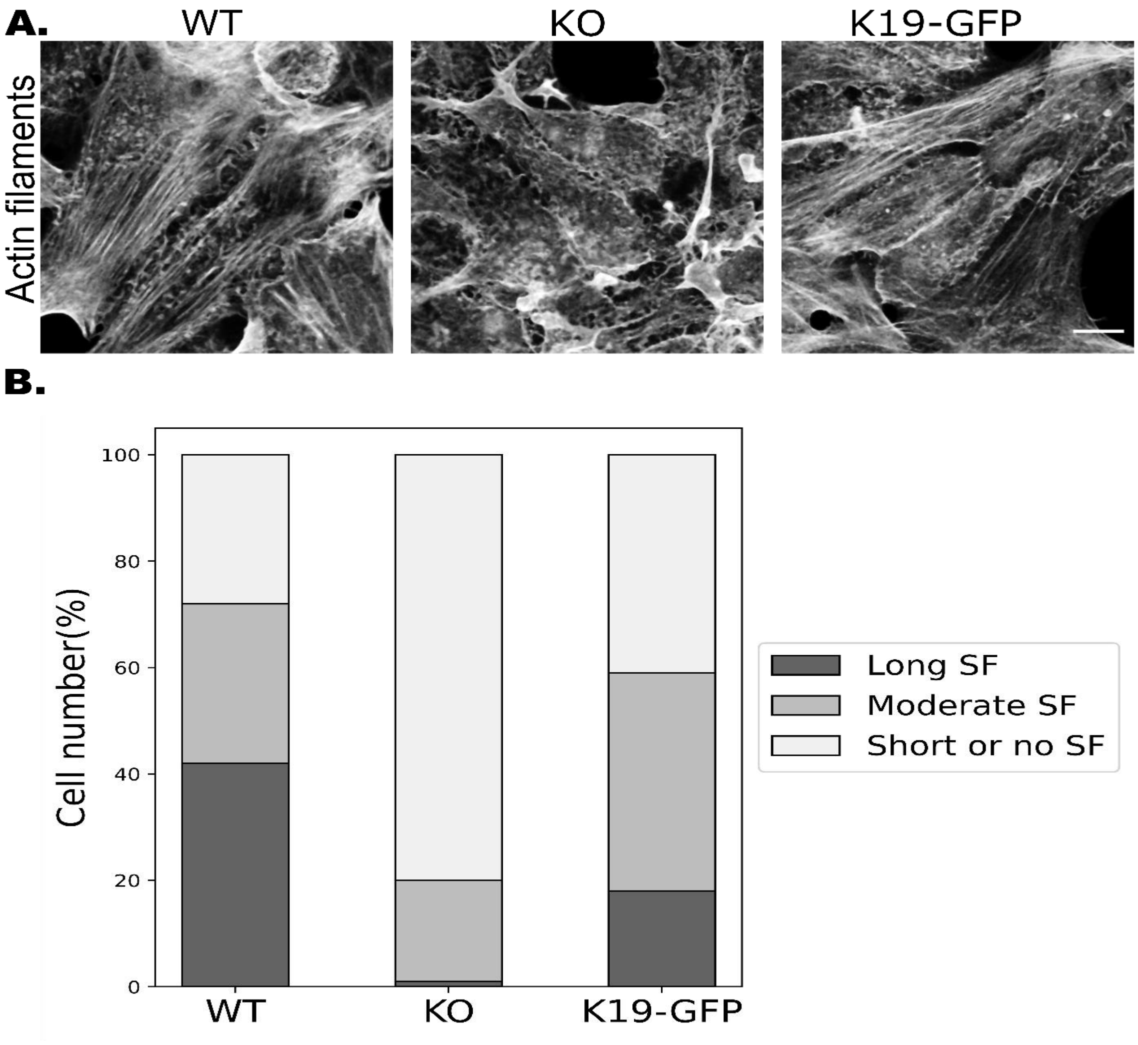

Figure S7. Absence of K19 eliminates force-induced actin stress fiber formation. (A) Confocal images of F-actin filaments in the WT, K19-KO, and K19-GFP rescued cell lines. Actin filaments were stained with Alexa568–phalloidin. Scale bar is 10 µm. **(B)** Quantitative analysis of actin stress fibers (SF) shows a significant reduction of longitudinal actin SFs, defined as thick, linear bundles aligned with the long axis of the cell, in the K19-KO cells compared to the WT and K19-GFP cells. Number of actin stress fibers (n) = 461 (WT), 280 (K19-KO), 460 (K19-GFP); Number of biological replicates (N) = 3. Cells were classified into three categories based on visual inspection of phalloidin-stained actin fibers: long, moderate, or short/no SF based on bundle length relative to the long axis of cell (≥75%, ~30–75%, <30%, respectively). The number of cells with long SF, moderate SF, and short or no SF was normalized by the total number of cells and shown as the percentage with respect to the total cell number in the plot.

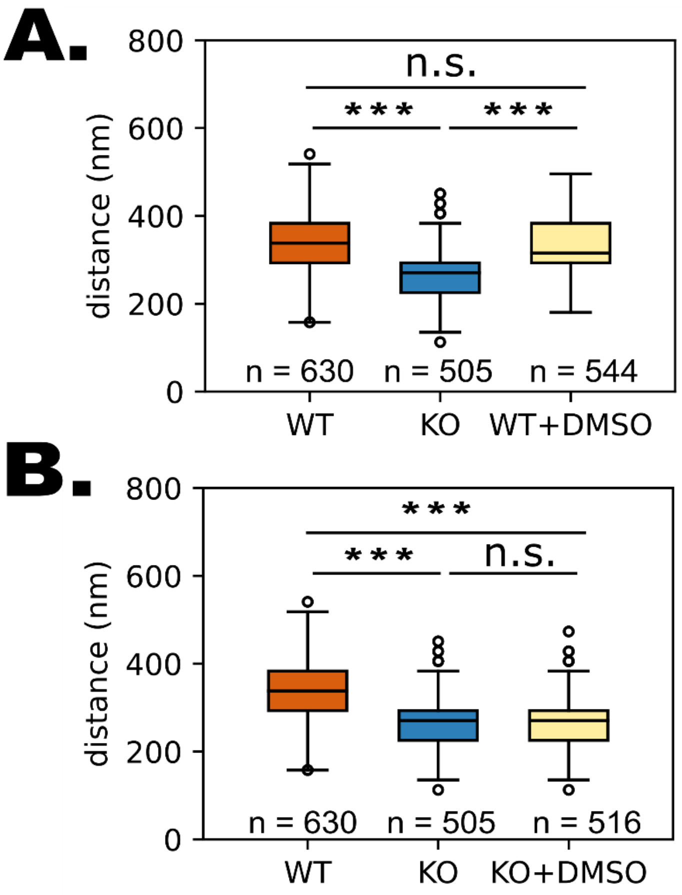

Figure S8. Quantitative analysis of desmosome width shows no statistically significant differences between the DMSO control group and the WT cells (A) and the K19-KO cells (B), respectively. Boxplots show the median, 25th, and 75th percentile with whiskers reaching the last data point within 1.5× interquartile range; dots indicate the outliers in the data; n = 630 (WT), 505 (K19-KO), 544 (WT+DMSO); n = 630 (WT), 505 (K19-KO), 516 (KO+DMSO); n represents the number of line scans across the desmosomes, N = 3 biological replicates. Kruskal-Wallis Test, followed by Dunn's multiple comparison Test with Holm adjustment; ***, P<0.001, ns, P>0.05. The detailed P values are provided in the Source data file.

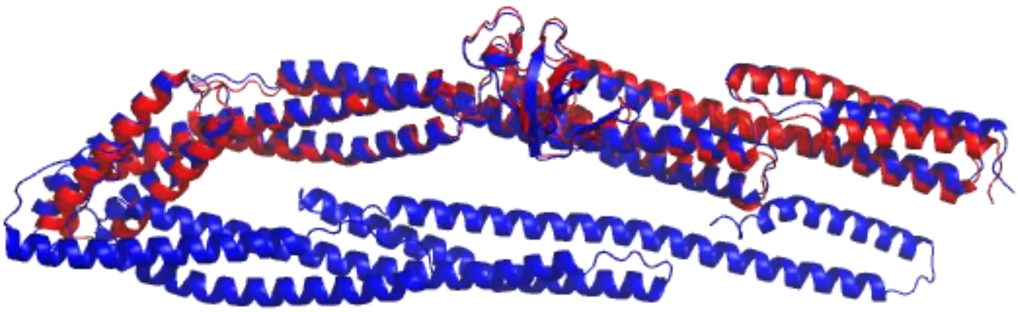

Figure S9. Structural characterization of DP plakin domain. Structural alignment between the crystal structure of the long arm of the DP plakin domain (red, PDB code 3R6N) and the AlphaFold generated DP plakin domain (blue) suggests that the AlphaFold prediction is close to the real structure of the DP plakin domain.

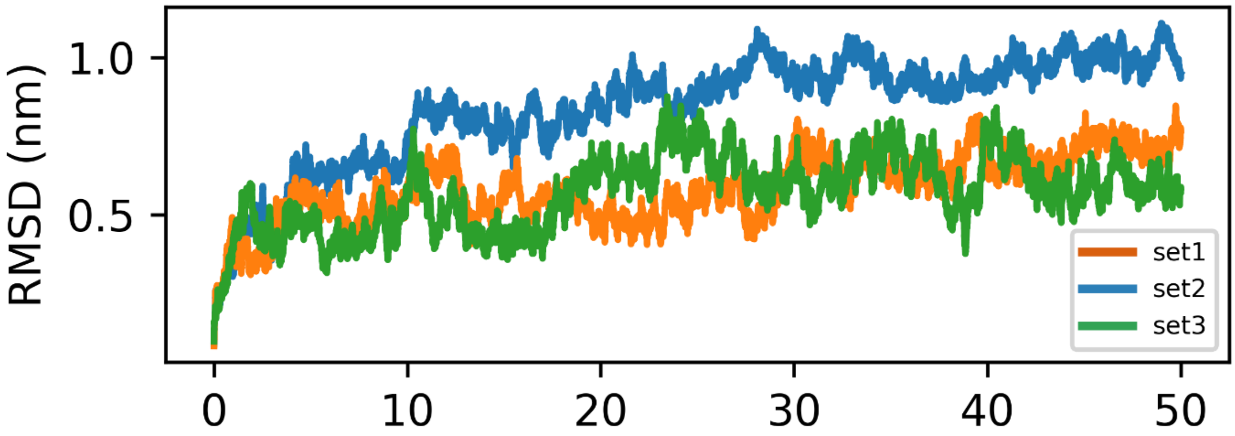

Figure S10. Protein backbone RMSD in MD simulations relative to the initial structures at the start of simulation. RMSD values were measured for three different sets. Stabilization of RMDS values within 10 ns suggest that structures are well equilibrated.

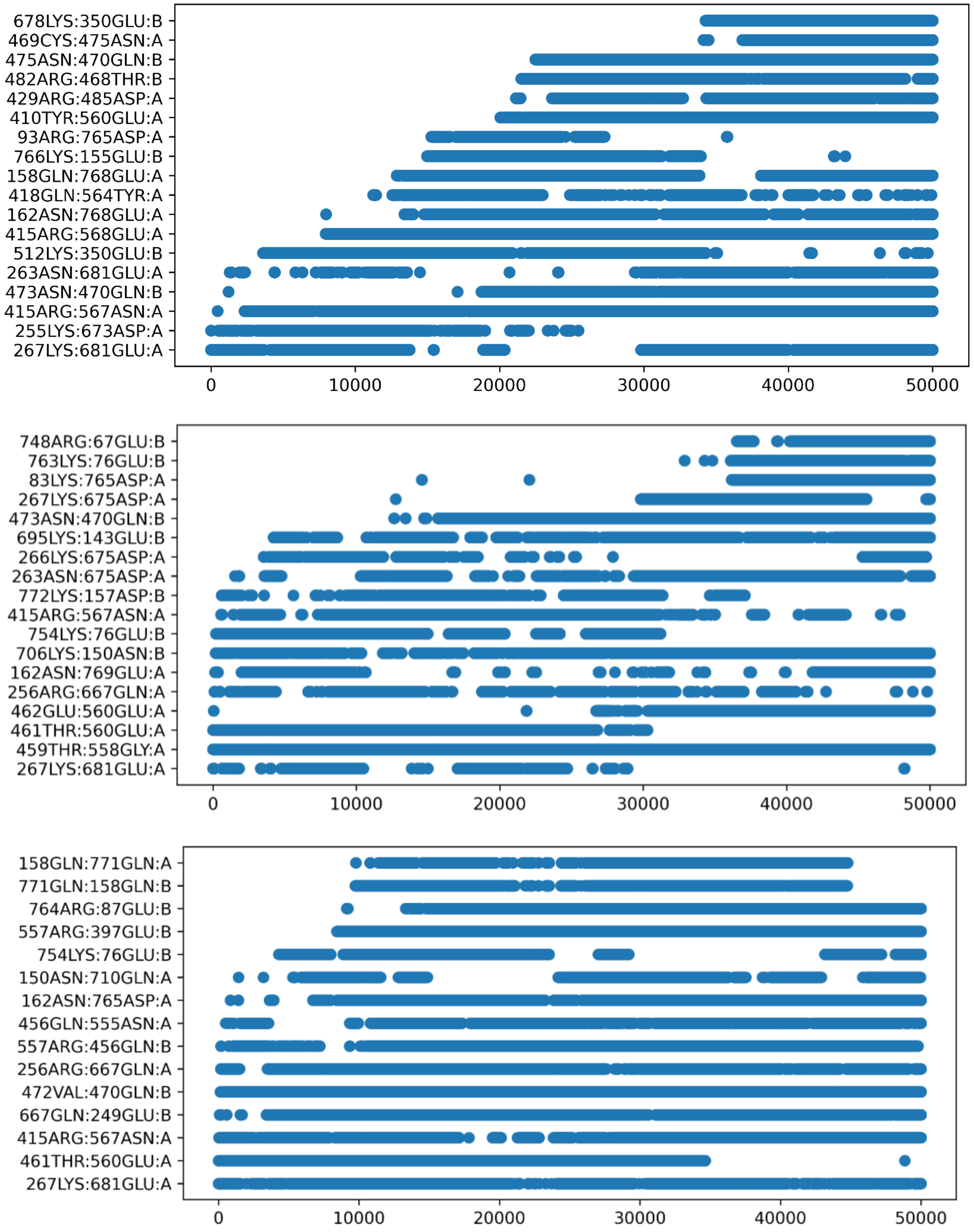

Figure S11. Persistent hydrogen bonds (i.e. hydrogen bonds that last >25% of the time) are observed between the long arm (SR3-6) and the short arm (SR7-8 and SR8-CT) of the DP plakin domain. Hydrogen bonds in set 1-3 are shown from top to bottom. Donor:acceptor pairs are labeled with "A", when hydrogen bond donor is from the long arm. Donor:acceptor pairs are labeled with "B", when hydrogen bond donor is from the short arm.

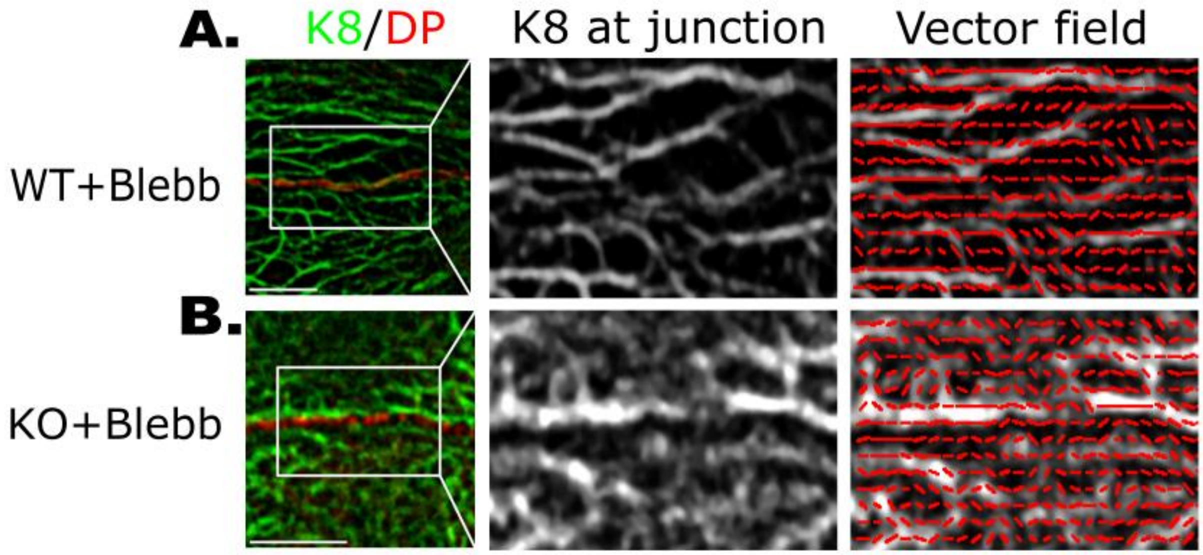

Figure S12. Keratin filaments reorient in response to reduced actomyosin forces with Blebbistatin treatment. (A-B) Representative confocal images of WT+Blebb **(A)** and KO+Blebb **(B)** cells immunolabeled for K8 (green) and DP (red). Scale bar: 5 µm (left panel). A magnified grayscale image highlights DP-associated K8 filaments at the junction with enhanced contrast (middle panel). The local dominant orientation of K8 filaments is represented as a vector field image (right panel). Experiments were done across three biological replicates.

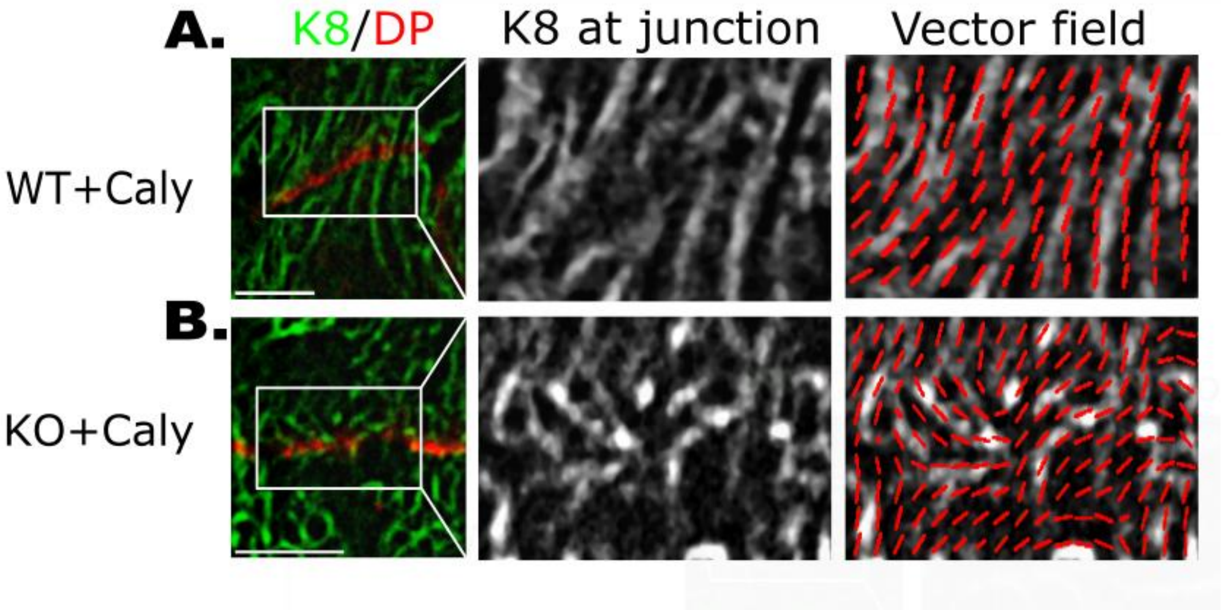

Figure S13. Keratin filaments reorient in response to increased actomyosin forces with Calyculin A treatment. (A-B) Representative confocal images of WT+Caly **(A)** and KO+Caly **(B)** cells immunolabeled for K8 (green) and DP (red). Scale bar: 5 μm (left panel). A magnified grayscale image highlights DP-associated K8 filaments at the junction with enhanced contrast (middle panel). The local dominant orientation of K8 filaments is represented as a vector field image (right panel). Experiments were done across three biological replicates.

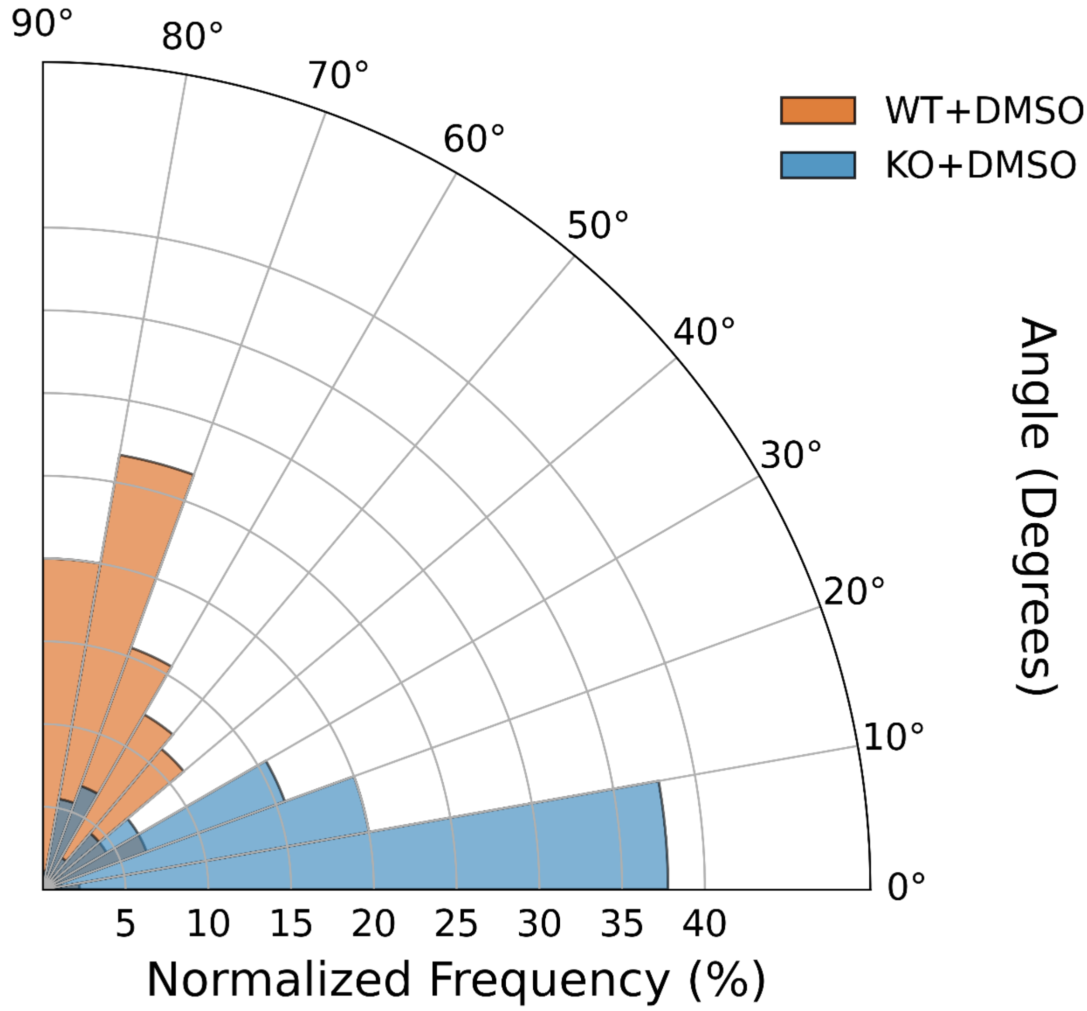

Figure S14. Quantitative analysis of the global dominant orientation of K8 filaments in WT and K19-KO cells treated with DMSO. Polar histogram of the global dominant orientation of K8 filaments in WT and K19-KO cells treated with DMSO. n = 90 (WT+DMSO), 90 (KO+DMSO); N = 3 biological replicates. Two-sided Mann-Whitney U test, $P < 0.001$. The detailed P value is provided in the Source data file. The K8 filaments in WT+DMSO cells exhibit a more radial organization, while filaments in KO+DMSO cells are horizontally aligned. The results show similar keratin organization patterns between DMSO-treated and untreated cells, with WT and WT+DMSO cells both displaying primarily radial filament organization, and KO and KO+DMSO cells exhibiting predominantly horizontal filament alignment.

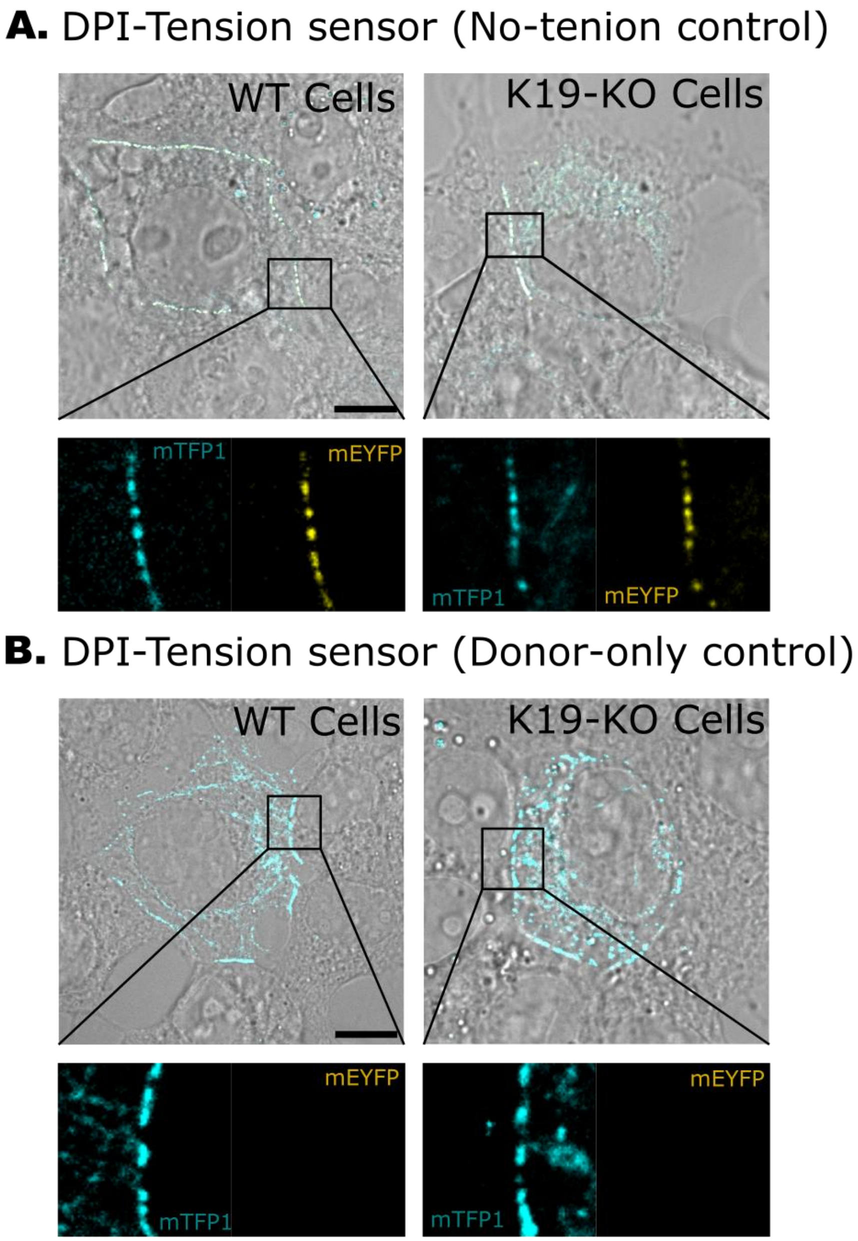

Figure S15. Representative images of WT and K19-KO cells transfected with DPI-no-tension control (A) and the donor-only control (B). The overlay of fluorescent signals of mTFP1 (cyan) and mEYFP (yellow) with the bright-field image shows the proper localization of DPI-no-tension control and DPI-donor-only control at the cell-cell contacts in both WT and K19-KO cells. Scale bar: 10 μm. The zoomed-in images reveal the formation of distinct DP puncta along the cell border. Experiments were done across three biological replicates.

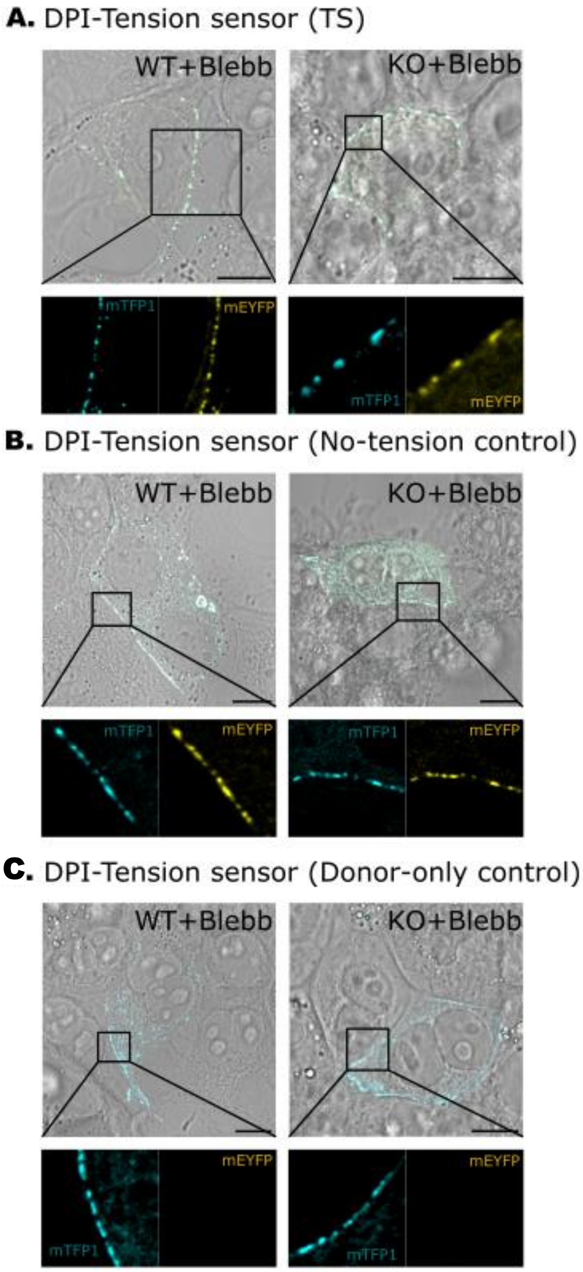

Figure S16. Representative images of WT+Blebb and K19-KO+Blebb cells transfected with DPI-TS (A), no-tension control (B), and the donor-only control (C). The overlay of fluorescent signals of mTFP1 (cyan) and mEYFP (yellow) with the bright-field image shows the proper localization of DPI-tension sensors, DPI-no-tension control, and DPI-donor-only control at the cell-cell contacts in both WT and K19-KO cells. Scale bar: 10 µm. The zoomed-in images reveal the formation of distinct DP puncta along the cell border. Experiments were done across three biological replicates.

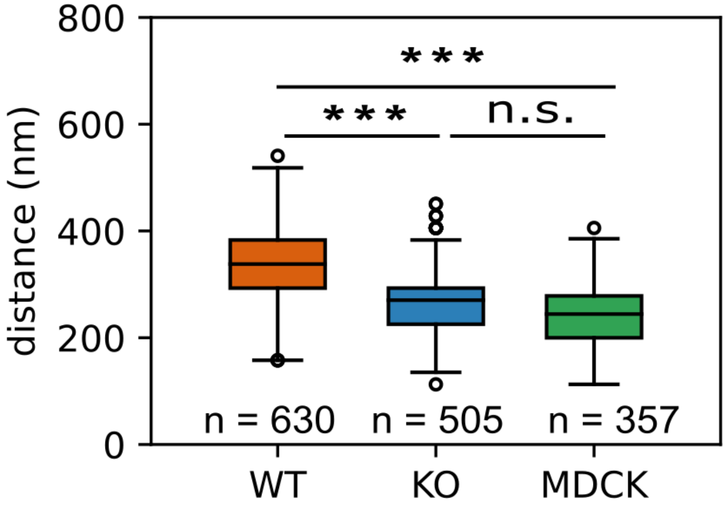

Figure S17. Desmosome width in MDCK cells is lower than WT cells and comparable to K19-KO cells. Boxplots show the median, 25th, and 75th percentile with whiskers reaching the last data point within 1.5× interquartile range; dots indicate the outliers in the data; n = 630 (WT), 505 (K19-KO), 357 (MDCK); n represents the number of line scans across the desmosomes. N = 3 biological replicates. Kruskal-Wallis Test, followed by Dunn's multiple comparison Test with Holm adjustment; ***, $P<0.001$; ns, $P>0.05$. The detailed P values are provided in the Source data file.

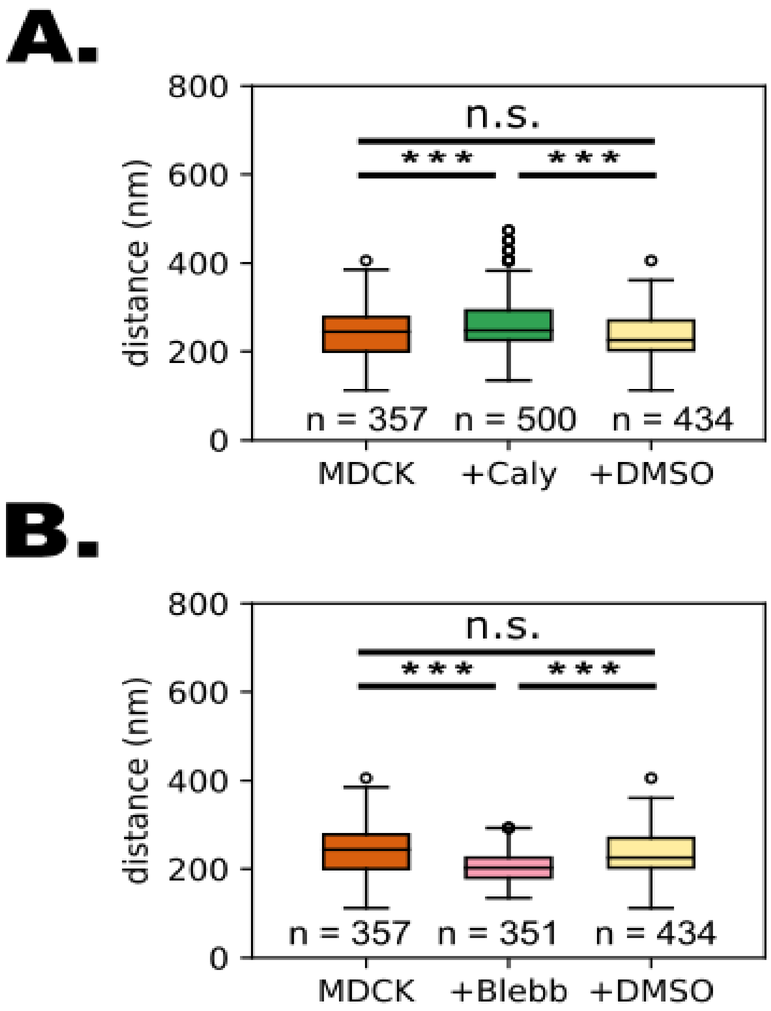

Figure S18. Desmosome widths in MDCK cells are altered in response to changes in actomyosin forces. (A) Quantification of desmosome width in MDCK cells with Calyculin A treatment**.** n = 357 (MDCK), 500 (+Caly), 434 (DMSO); N = 3 biological replicates. Kruskal-Wallis Test, followed by Dunn's multiple comparison Test with Holm adjustment; ***, P<0.001; ns, P>0.05. Desmosome width in MDCK cells becomes significantly higher upon increased actomyosin contractility**. (B)** Quantification of desmosome width in MDCK cells with Blebbistatin treatment. n = 357 (MDCK), 351 (+Caly), 434 (DMSO); N = 3 biological replicates. Two-sided Mann-Whitney's U test; ***, P<0.001; ns, P>0.05. Desmosome width in MDCK cells becomes significantly lower upon reduced actomyosin contractility. Boxplots show the median, 25th, and 75th percentile with whiskers reaching the last data point within 1.5× interquartile range; dots indicate the outliers in the data. The number of data points n represents the number of line scans across the desmosomes. The detailed P values are provided in the Source data file.

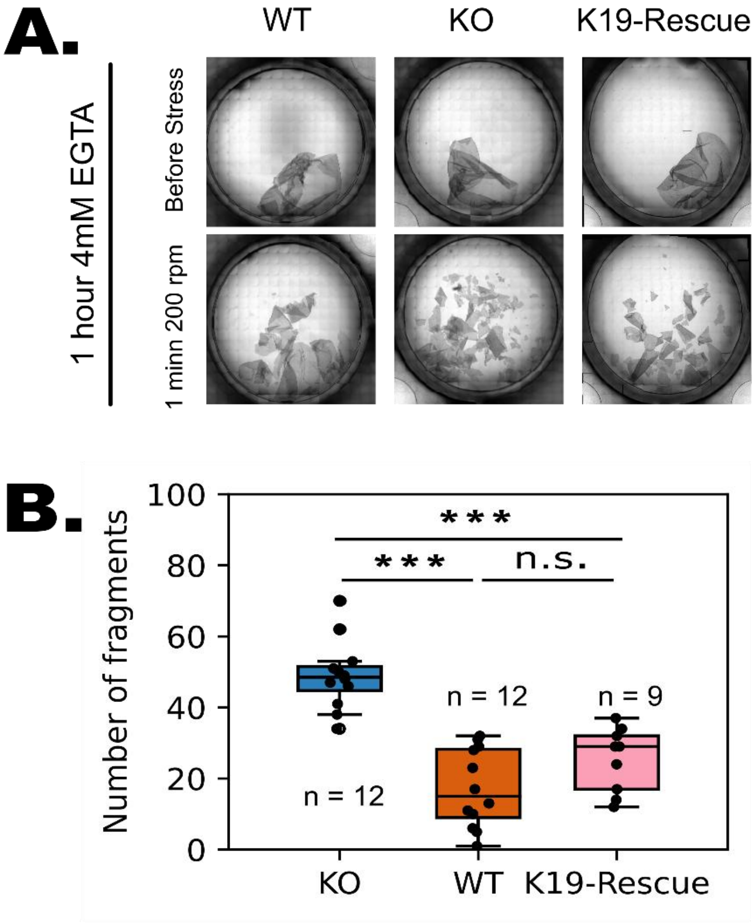

Figure S19. Desmosome widening is not indicative of reduced adhesion strength. (A) Dispase assay after 24 h of plating. The confluent cell sheets are treated with 4 mM EGTA for 1 h. The images show the intact cell sheets for WT, K19-KO, and K19-GFP rescued cells before stress and fragmented cell sheets after applying mechanical stress. **(B)** Quantification of the dispase assay from K19-KO, WT, and K19-GFP cells. n = 12 (KO), 12 (WT), 9 (K19-GFP); n represents the number of cell sheets, N = 3 biological replicates. Two-sided Mann-Whitney's U test; ***, $P<0.001$; ns, $P>0.05$. The K19-KO cell sheets show a greater number of fragments compared to WT cell sheets, while K19-GFP cell sheets generate a similar number of fragments compared to WT. Boxplots show the median, 25th, and 75th percentile with whiskers reaching the last data point within 1.5× interquartile range; dots indicate the outliers in the data. The detailed P values are provided in the Source data file.

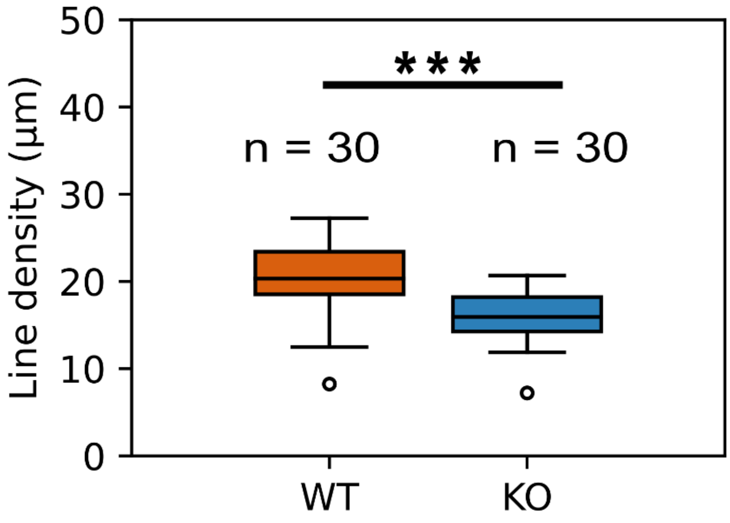

Figure S20. The DP line density is marginally higher in the WT cells than the K19-KO cells. The line density is defined as the desmosome length along the cell border normalized by number of cells. Number of datapoints (n) = 30 (WT), 30 (K19-KO); n represents the number of images, Number of biological replicates (N) = 3. Two-sided Mann-Whitney's U test; ***, P<0.001. Boxplots show the median, 25th, and 75th percentile with whiskers reaching the last data point within 1.5× interquartile range; dots indicate the outliers in the data. The detailed P values are provided in the Source data file.

A. **B.**

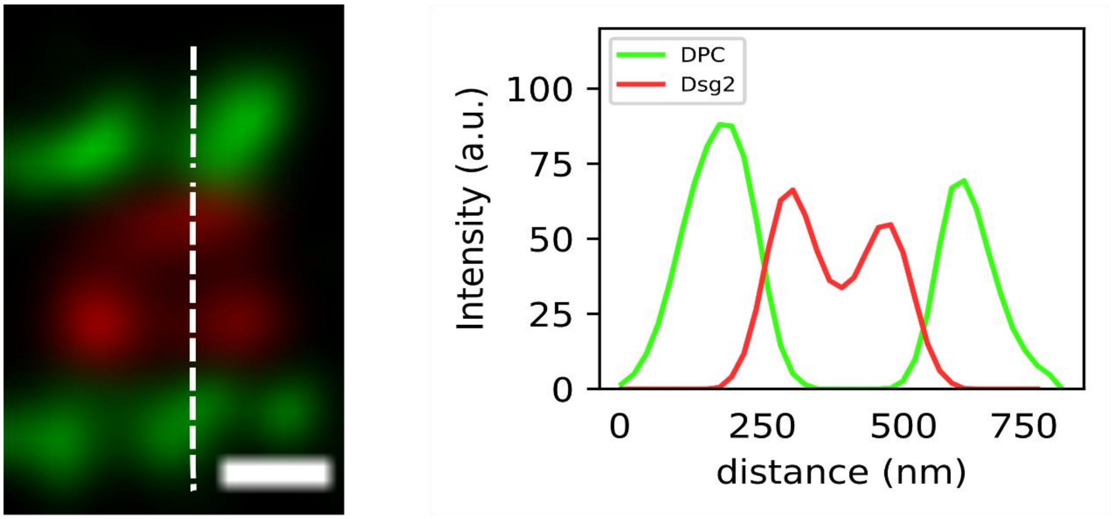

Figure S21. Representative STED image of broken desmosomes in WT cells. (A) DP railroad track immunolabeled for DPC (green) and Dsg2 (red). The Dsg2 signals are resolved (i.e., spatially separated) in the image. The scale bar is 200 nm. **(B)** Line-scan analysis of DPC and Dsg2 fluorescence intensity (indicated in A).
