# Supplementary material for: Actomyosin forces trigger a conformational change in desmoplakin within desmosomes
Source: Nat Commun. 2025 Oct 10;16:9052. doi: 10.1038/s41467-025-64124-4 (PMC12514153; doi:10.1038/s41467-025-64124-4)
Supplement: Supplementary file 3 — Description of Additional Supplementary Files [file 41467_2025_64124_MOESM3_ESM.pdf]

Description of Additional Supplementary Files

File Name: Supplementary Movie 1
Description: An example of constant-force SMD simulation, related to Fig. 4. The constant-force SMD simulation illustrates the conversion of the DPN plakin domain from a folded (closed) to an extended (open) conformation upon pulling, which accounts for the elongation of 30-33 nm of the DP plakin domain. Color scheme: SR3-4 (green), SH3 (magenta), SR5-6 (blue), SR7-8 (orange), and SR8-CT (cyan).
