# Supplementary material for: Actomyosin forces trigger a conformational change in desmoplakin within desmosomes
Source: Nat Commun. 2025 Oct 10;16:9052. doi: 10.1038/s41467-025-64124-4 (PMC12514153; doi:10.1038/s41467-025-64124-4)
Supplement: Supplementary file 5 — Reporting Summary [file 41467_2025_64124_MOESM5_ESM.pdf]

nature portfolio

Corresponding author(s): Sanjeevi Sivasankar

Last updated by author(s): Aug 26, 2025

Reporting Summary

Nature Portfolio wishes to improve the reproducibility of the work that we publish. This form provides structure for consistency and transparency in reporting. For further information on Nature Portfolio policies, see our Editorial Policies and the Editorial Policy Checklist.

Statistics

For all statistical analyses, confirm that the following items are present in the figure legend, table legend, main text, or Methods section.

n/a	Confirmed	
☐	☒	The exact sample size (*n*) for each experimental group/condition, given as a discrete number and unit of measurement
☐	☒	A statement on whether measurements were taken from distinct samples or whether the same sample was measured repeatedly
☐	☒	The statistical test(s) used AND whether they are one- or two-sided<br>*Only common tests should be described solely by name; describe more complex techniques in the Methods section.*
☒	☐	A description of all covariates tested
☐	☒	A description of any assumptions or corrections, such as tests of normality and adjustment for multiple comparisons
☐	☒	A full description of the statistical parameters including central tendency (e.g. means) or other basic estimates (e.g. regression coefficient) AND variation (e.g. standard deviation) or associated estimates of uncertainty (e.g. confidence intervals)
☐	☒	For null hypothesis testing, the test statistic (e.g. *F*, *t*, *r*) with confidence intervals, effect sizes, degrees of freedom and *P* value noted<br>*Give P values as exact values whenever suitable.*
☒	☐	For Bayesian analysis, information on the choice of priors and Markov chain Monte Carlo settings
☒	☐	For hierarchical and complex designs, identification of the appropriate level for tests and full reporting of outcomes
☒	☐	Estimates of effect sizes (e.g. Cohen's *d*, Pearson's *r*), indicating how they were calculated

Our web collection on statistics for biologists contains articles on many of the points above.

Software and code

Policy information about availability of computer code

Data collection	Western blots data was acquired using Image Lab from Bio-Rad or iBright™ CL1500 Imaging System from Invitrogen . Molecular Dynamics simulations were performed using Gromacs 2020.1. All confocal imaging data was acquired using Leica Stellaris confocal microscope platform. All STED and FRET-FLIM imaging was acquired using Leica TCS SP8 STED 3x confocal miscroscope platform.
Data analysis	Molecular dynamics simulations were visualized with VMD 1.9.3, figures were created with PyMOL open-source 1.8.4.0, and RMSDs were calculated using Gromacs 2020.2. Imaging data was analyzed using image J. The following quantitative analysis after imaging was analyzed using Excel 2020 (Microsoft), MATLAB R2022B, and Python3. Fluorescence lifetime data were analyzed using the built-in FLIM Wizard in LAS X software. All statistical tests were performed using Python custom code or R studio.

For manuscripts utilizing custom algorithms or software that are central to the research but not yet described in published literature, software must be made available to editors and reviewers. We strongly encourage code deposition in a community repository (e.g. GitHub). See the Nature Portfolio guidelines for submitting code & software for further information.

Data

Policy information about availability of data

All manuscripts must include a data availability statement. This statement should provide the following information, where applicable:

- Accession codes, unique identifiers, or web links for publicly available datasets
- A description of any restrictions on data availability
- For clinical datasets or third party data, please ensure that the statement adheres to our policy

All data produced by this research have been made available in the manuscript and supporting information. Simulation input files and a coordinate file of the final output have been deposited in a public repository. Custom code has been deposited in a public repository.

Research involving human participants, their data, or biological material

Policy information about studies with human participants or human data. See also policy information about sex, gender (identity/presentation), and sexual orientation and race, ethnicity and racism.

Reporting on sex and gender	*Use the terms sex (biological attribute) and gender (shaped by social and cultural circumstances) carefully in order to avoid confusing both terms. Indicate if findings apply to only one sex or gender; describe whether sex and gender were considered in study design; whether sex and/or gender was determined based on self-reporting or assigned and methods used.* *Provide in the source data disaggregated sex and gender data, where this information has been collected, and if consent has been obtained for sharing of individual-level data; provide overall numbers in this Reporting Summary. Please state if this information has not been collected.* *Report sex- and gender-based analyses where performed, justify reasons for lack of sex- and gender-based analysis.*
Reporting on race, ethnicity, or other socially relevant groupings	*Please specify the socially constructed or socially relevant categorization variable(s) used in your manuscript and explain why they were used. Please note that such variables should not be used as proxies for other socially constructed/relevant variables (for example, race or ethnicity should not be used as a proxy for socioeconomic status).* *Provide clear definitions of the relevant terms used, how they were provided (by the participants/respondents, the researchers, or third parties), and the method(s) used to classify people into the different categories (e.g. self-report, census or administrative data, social media data, etc.)* *Please provide details about how you controlled for confounding variables in your analyses.*
Population characteristics	*Describe the covariate-relevant population characteristics of the human research participants (e.g. age, genotypic information, past and current diagnosis and treatment categories). If you filled out the behavioural & social sciences study design questions and have nothing to add here, write "See above."*
Recruitment	*Describe how participants were recruited. Outline any potential self-selection bias or other biases that may be present and how these are likely to impact results.*
Ethics oversight	*Identify the organization(s) that approved the study protocol.*

Note that full information on the approval of the study protocol must also be provided in the manuscript.

Field-specific reporting

Please select the one below that is the best fit for your research. If you are not sure, read the appropriate sections before making your selection.

☒ Life sciences ☐ Behavioural & social sciences ☐ Ecological, evolutionary & environmental sciences

For a reference copy of the document with all sections, see nature.com/documents/nr-reporting-summary-flat.pdf

Life sciences study design

All studies must disclose on these points even when the disclosure is negative.

Sample size	The total number of all desmosome plaque-to-plaque distance measurements performed was at least 300 line scans across the desmosome complex in each condition. The total number of keratin filament orientation analysis was at least 90 for each condition. The total number of FRET-FLIM measurements was 30 images for each condition and for each image 20–40 ROIs were manually selected, focusing on individual DP puncta.
Data exclusions	For the desmosome distance measurement analysis, we eliminated the ruptured desmosomes that have two peaks observed for the Dsg2 signals (Figure S21), from further analysis, and only focused on intact desmosomes with unresolvable Dsg2 signals.
Replication	For molecular dynamics simulations, three simulations per simulation conditions were performed. Cardiomyocytes were isolated from 20-24 pups total from two separate preps All other cell-related experiments were performed with three biological replicates.

Randomization	Experimental groups were defined by genetic background (WT vs. K19-KO) and treatment conditions (e.g., drug treatments vs. control). Allocation are therefore determined by experimental design. All conditions were processed in parallel using identical procedures to minimize technical variability, and replicates across groups were collected under the same imaging and analysis conditions. Fluorescence imaging data was randomly selected from a 22 mm * 22 mm cover slips.
Blinding	All imaging data were acquired randomly from the samples. After image acquisition, quantitative assessments were conduced using custom-made Image J macros and Python script and standardized parameters to ensure consistency across samples. FRET-FLIM data were acquired using identical laser excitation parameters, photon counting thresholds, and acquisition settings across all samples. Lifetime fitting and FRET efficiency calculations were performed with the predefined analysis parameters across all datasets.

Reporting for specific materials, systems and methods

We require information from authors about some types of materials, experimental systems and methods used in many studies. Here, indicate whether each material, system or method listed is relevant to your study. If you are not sure if a list item applies to your research, read the appropriate section before selecting a response.

Materials & experimental systems

n/a	Involved in the study	
☐	☒	Antibodies
☐	☒	Eukaryotic cell lines
☒	☐	Palaeontology and archaeology
☐	☒	Animals and other organisms
☒	☐	Clinical data
☒	☐	Dual use research of concern
☒	☐	Plants

Methods

n/a	Involved in the study	
☒	☐	ChIP-seq
☒	☐	Flow cytometry
☒	☐	MRI-based neuroimaging

Antibodies

Antibodies used

Primary antibodies for immunofluorescence imaging:
Anti-Dsg2 : Supplier- R&D systems, Catalog number – MAB947
Anti-Dsg2 : Supplier- Proteintech, Catalog number – 21880-1-AP
Anti-DPC : Supplier- Bethyl Lab, Catalog number – A303-356A
Anti-DPN : Supplier- Proteintech, Catalog number – 25318-1-AP
Anti-K8: Supplier- ThermoFisher, Catalog number – MA1-06318
Anti-K18: Supplier- ThermoFisher, Catalog number – MA1-19047
Anti-GFP: Supplier- Rockland, Catalog number – 600-901-215
Phalloidin: Supplier-Invitrogen , Catalog number – AF-488
Phalloidin: Supplier-Invitrogen , Catalog number – AF-568

Secondary antibodies for immunofluorescence imaging:
Goat anti-Chicken IgY (H+L)-Alexa Fluor 488, Supplier- Invtrogen, Catalog number- A-11039
Goat anti-mouse IgY (H+L)-Alexa Fluor 594, Supplier- Invtrogen, Catalog number- A-11005
Goat anti-rabbit IgY (H+L)-Alexa Fluor 647, Supplier- Invtrogen, Catalog number- A-21245

Primary antibodies for western blot and Co-IP experiments:
Anti-actin : Supplier- Proteintech, Catalog number – 66009-1-Ig
Anti-K8 : Supplier- Proteintech, Catalog number – 17514-1-AP
Anti- K18: Supplier- Proteintech, Catalog number – 10830-1-AP
Anti-Dsg2 : Supplier- Proteintech, Catalog number – 21880-1-AP
Anti-plectin : Supplier- Proteintech, Catalog number – 29170-1-AP
Anti- desmoplakin: Supplier- Proteintech, Catalog number – 25318-1-AP
Anti- tubulin: Supplier- Proteintech, Catalog number – 66031-1-Ig
Anti-K19 : Supplier- Santa Cruz Biotechnology, Catalog number – A53-B/A2
Anti-plakophilin-3 : Supplier- Santa Cruz Biotechnology , Catalog number – E-10
Anti-plakophilin-1 : Supplier- Santa Cruz Biotechnology , Catalog number – 10B2
Anti-desmocolin-1 : Supplier- Santa Cruz Biotechnology, Catalog number – A-4
Anti- desmocolin-2/3 : Supplier- Santa Cruz Biotechnology, Catalog number – 7G6
Anti- myosin-10 : Supplier- Santa Cruz Biotechnology, Catalog number – A-3
Control IgG: Supplier- Santa Cruz Biotechnology, Catalog number – sc-2025

Secondary antibodies for western blot and Co-IP experiments:
Goat Anti-Mouse IgG (H+L)-HRP Conjugate, Supplier- BioRad, Catalog number- 1706516
Goat Anti-Mouse IgG (whole molecule)-HRP Conjugate, Supplier- Sigma Aldrich, Catalog number- A4416
Goat Anti-Rabbit IgG (whole molecule)-HRP Conjugate, Supplier- Sigma Aldrich, Catalog number- A6154

Validation | All the antibodies were purchased from commercial vendors and were validated by the manufacturer.

Eukaryotic cell lines

Policy information about cell lines and Sex and Gender in Research

Cell line source(s)
MCF7 WT cells and MDCK cells were purchased from ATCC. The K19-KO cells were previously generated using the CRISPR/Cas9 technology as described in the Methods section. We developed the K19-GFP rescue cell line following protocols described in the method. Primary cardiomyocytes were isolated from Swiss Webster mice (Taconic Biosciences) as described in the Methods section.

Authentication
The rescued cell line was verified using Western Blots (Figure S2).

Mycoplasma contamination
These cell lines were not tested for mycoplasma contamination.

Commonly misidentified lines
(See ICLAC register)
There were no commonly misidentified cell lines used in this study.

Animals and other research organisms

Policy information about studies involving animals; ARRIVE guidelines recommended for reporting animal research, and Sex and Gender in Research

Laboratory animals
Swiss Webster outbred mice were the source of all primary neonatal cardiomyocytes.

Wild animals
Provide details on animals observed in or captured in the field; report species and age where possible. Describe how animals were caught and transported and what happened to captive animals after the study (if killed, explain why and describe method; if released, say where and when) OR state that the study did not involve wild animals.

Reporting on sex
Hearts from male and female mouse pups were isolated and combined during the prep. Thus, sex was not a biological variable in the primary cell culture experiments.

Field-collected samples
For laboratory work with field-collected samples, describe all relevant parameters such as housing, maintenance, temperature, photoperiod and end-of-experiment protocol OR state that the study did not involve samples collected from the field.

Ethics oversight
All animal work was approved by the University of Pittsburgh Division of Laboratory Animal Resources.

Note that full information on the approval of the study protocol must also be provided in the manuscript.

Plants

Seed stocks
Report on the source of all seed stocks or other plant material used. If applicable, state the seed stock centre and catalogue number. If plant specimens were collected from the field, describe the collection location, date and sampling procedures.

Novel plant genotypes
Describe the methods by which all novel plant genotypes were produced. This includes those generated by transgenic approaches, gene editing, chemical/radiation-based mutagenesis and hybridization. For transgenic lines, describe the transformation method, the number of independent lines analyzed and the generation upon which experiments were performed. For gene-edited lines, describe the editor used, the endogenous sequence targeted for editing, the targeting guide RNA sequence (if applicable) and how the editor was applied.

Authentication
Describe any authentication procedures for each seed stock used or novel genotype generated. Describe any experiments used to assess the effect of a mutation and, where applicable, how potential secondary effects (e.g. second site T-DNA insertions, mosiacism, off-target gene editing) were examined.
